# Psychological Distress and Social Functioning in Elderly Spanish People: A Gender Analysis

**DOI:** 10.3390/ijerph16030341

**Published:** 2019-01-26

**Authors:** M. Pilar Matud, M. Concepción García

**Affiliations:** Department of Clinical Psychology, Psychobiology and Methodology, Universidad de La Laguna, 38205 La Laguna, Spain; extmgarciaa@ull.edu.es

**Keywords:** psychological distress, social functioning, elderly, gender, stress, coping styles, social support, self-esteem

## Abstract

Psychological distress has been considered a key component in the psychosocial functioning and functional disability of the elderly, but the determining factors of social functioning and psychological distress in the elderly people are not yet fully known. The aim of this study is to perform a gender analysis of the relevance of psychological distress and psychosocial factors in the social functioning of the elderly. A cross-sectional study with a sample of 589 men and 684 women from the general Spanish population aged between 65 and 94 years was conducted. All participants were evaluated through questionnaires and scales that assess psychological distress, social functioning, stress, coping styles, self-esteem and social support. Results: Women scored higher than men in psychological distress, chronic stress, emotional coping and instrumental social support, whereas men scored higher than women in self-esteem and rational coping. Psychological distress was significantly associated in women and men with worse social functioning, which was also lower in older people and in women with lower self-esteem. Psychological distress has a considerable impact on the social functioning of the elderly, and gender is a relevant factor in the psychological distress experienced and its predictors.

## 1. Introduction

The world population is aging. Worldwide, the proportion of people older than 59 years is growing faster than any other age group. This trend is also evident in Spain, where the expectations about the proportion of people aged 60 and over are 31.4% by 2025 [1]. Aging results from the interaction of the processes that occur over time, the interactions of time, genetics, disease, and environmental and behavioral factors [2]; it is also associated with numerous physical, physiologic, emotional, and cognitive changes and decrease functions, although there is no prescribed pattern for their order of appearance [3]. “When an individual ages, their reduced reserve capacity makes them more vulnerable to stresses and places an older individual at greater risk of succumbing to stresses that a younger patient might overcome” [2] (p. 467). In the late 1990s, the World Health Organization adopted the term “active ageing”, which aims to extend people´s healthy life expectancy and quality of life during aging [1] and refers to “the process of optimizing opportunities for health, participation and security in order to enhance quality of life as people age” (p. 12). 

The accumulated evidence indicates that the effects of psychosocial stress are adverse for health, most notably if demanding and repeated [4]. A variety of psychosocial stressors can hinder the health-related quality of life of the elderly [5], and biological, social and economic stressors may increase the risk of psychological distress in older adults [6]. Research linking stress, health and aging differentiates two approaches: the life event tradition, which focuses on the molar impact of major life changes that require significant adjustment by the individual, and a second one that focuses on chronic stressors and the recurrent or persistent difficulties of life that may increase exposure to daily hassles [7]. Evidence suggests that excessive or accumulated stress can accelerate epigenetic aging [8], contribute to autonomic dysregulation [9], impact the immune system through the secretion of hormones, which are also modified during aging [10], and adversely affect cognitive functions [11,12]. The brain is the central organ of stress and adaptation to stressors [13]; research has acknowledged that stress has negative effects on the brain across lifespan, but their scope is greater in early and late life [14]. Although many studies have established some associations between stress and health-related outcomes, people do not always present the same effects. There are individual differences in the stress processes, which are influenced by several factors, including socioeconomic status, personal dispositions, and life transition [7]. While the stress processes are not yet fully known, since the 80s it was generally accepted that exposure to stress alone does not directly lead to health problems, but its effects are mediated and moderated by other variables such as coping and social support [15]. According to Folkman [16] (p. 902) “coping refers to the thoughts and behaviors people use to manage the internal and external demands of stressful events”. Coping includes behaviors and strategies that are generally grouped into two kinds, problem-focused coping and emotion-focused coping. In the first case, strategies and behaviors such as information gathering and decision making are aimed at solving the problem causing the discomfort; emotion-focused coping rather deals with the regulating of negative emotions by using strategies such as distancing, escape-avoidance and the search of emotional support [16]. Emotion regulation and social support are two constructs that contribute to resilience and have specific patterns in older adults [17].

In addition to the psychosocial stressors and the losses occurring in late life, the risk of distress is greater as frailty and physical illnesses increase [18]. Psychological distress is a widespread indicator of mental illness and mental health in clinical settings, in research, and in public health [19]. Moreover, it has been related to increasing rates of death from several major causes, such as cerebral disease, cardiovascular disease, cancer and deaths from external causes [20,21]; recent research has likewise reported that psychological distress raises the risk of developing some diseases such as arthritis, cardiovascular disease, diabetes, and chronic obstructive pulmonary disease [22]. Psychological distress has been considered a key component in the psychosocial functioning of older people [23]; in the case of the elderly, it may occur as a consequence of poorer cognitive functioning [24]. Actually, research has reported that psychological distress was associated with and increased the risk of functional disability in the elderly [25,26].

Demographic, social and personal factors like gender, socioeconomic status, age, marital status, social support, and self-esteem have been associated with psychological distress [18,27,28,29,30,31,32,33,34]. Psychological distress has been negatively associated with age and with self-esteem [30]. Besides, in elderly people, psychological distress has been associated with less social support [28], whereas the risk of psychological distress has proved lower in the case of higher education, higher income and married status [18,34]. Studies carried out in several countries have realized gender differences in psychological distress, as women presented a higher mean level than men [19,28,32,34,35,36]. But gender differences in psychological distress seem to be influenced by sociodemographic and occupational variables and the disparities between women and men in psychological distress may diminish or even disappear in case of a high occupational level [37]. In addition, such gender differences may vary depending on the context of the study [38]. Another factor that may be relevant when approaching gender differences in psychological distress is stress and coping styles. Although the existence of gender differences in stress may depend on the type of stressor, it has been found that women report more chronic stress than men [35,36,39]. The results of the research on gender differences in coping have not been conclusive, but some studies indicate that emotion-focused coping is more common in women than in men, and problem-focused coping more common in men [36,39]. Emotion-focused coping is associated with greater psychological distress whereas psychological distress provides lower levels in the case of problem-focused coping [36,39,40,41]. Gender differences in psychological distress are an important clinical and public health issue, and further researches are needed to know the factors underlying these differences [19].

Research has by and large concluded that there are resources influencing indirectly and negatively on psychological distress, and stressors whose effect on distress is direct and positive. However, the dynamic mechanisms involved in the relationship among stressors, resources and psychological distress are not yet clear [6]. Studies have not generally approached the relevance of gender in such processes. Gender is acknowledged an important social determinant of health [42]; in fact, the WHO in its “Ageing and Health” Programme highlights the importance of recognizing gender differences [1]. The main aim of the present study has been to perform a gender-focused analysis so as to examine the relevance of psychological distress and psychosocial factors in the social functioning of the elderly. The specific objectives of the work are three: (1) To know the relevance of gender in psychological distress and social functioning of the elderly. (2) To know the relevance of the sociodemographic variables, psychosocial stress and personal and social resources (coping styles, self-esteem and social support) in the psychological distress of elderly individuals. (3) To know the relevance of sociodemographic variables, psychosocial stress, psychological distress and personal and social resources (coping styles, self-esteem and social support) in the social functioning of aged people. The specific hypotheses for this research are as follows:

(1) Women will present greater psychological distress than men.
(2) Greater stress will be associated with greater psychological distress.
(3) Greater stress, greater psychological distress and older age will be associated with worse social functioning.

## 2. Materials and Methods

### 2.1. Participants

The sample consisted of 589 men and 684 women from the Spanish general population whose ages ranged between 65 and 94 years (*M* = 71.43, *SD* = 5.81). Table 1 shows the main sociodemographic characteristics of the sample. Women and men did not differ in age or in number of children, but there were statistically significant differences in educational level and marital status. Although there was considerable diversity in the education levels of women and men, most often they had only completed elementary studies, which was more common among women (48.4%) than among men (39.2%). Men did more frequently than women complete high school or 5-year university degrees, while it was more common for women to have completed 3-year university degrees. With regard to the marital status, it also showed diversity. Most often, subjects were married or lived as partners, although men rated higher (78.8%) than women (55.3%). The rate for widowed women (30.8%) was higher than in the case of widowers (9.5%). The figures for childless men were 10.4% and 8.3% for women. The range of the number of children was between 0 and 8 in the men subsample and between 0 and 9 in that of women. Most people no longer worked, except 9.8% of men and 7.6% of women, who had different occupations. There were no statistically significant differences between women and men in terms of occupation, χ^2^(5, *N* = 91) = 2.04, *p* = 0.84.

### 2.2. Measures

#### 2.2.1. Dependent Variables: Psychological Distress and Social Functioning

Psychological distress and social functioning were assessed by using the subscales of the QHQ-28 [43], a self-report instrument that assesses current and recent complaints and is widely used to measure the health status of individuals [44]. The GHQ includes 20 “negative” items that address distressing symptoms and 8 “positive” items which represent the ability to carry out normal functions [44,45]. Although the GHQ-28 has not usually been used to test positive attributes but only the absence of distress, more innovative approaches have positively encoded the responses to the positively worded GHQ items and have used the items of the social dysfunction subscale of the GHQ-28 to assess positive social functioning [44].

Psychological distress was assessed by using the subscales of severe depression, anxiety and insomnia, and somatic symptoms of the GHQ-28 [43]. An example item is “Been thinking of yourself as a worthless person?”. Items were scored according to the Likert-type scale that assigns a weight to each score, from 0 (less than usual) to 3 (much more than usual), so higher scores indicate a higher level of psychological distress. For the current sample, the 21 items of the three scales were grouped into a factor whose Cronbach’ alpha was 0.93.

Participants’ social functioning was assessed drawing on the seven items of the social dysfunction subscale of the GHQ-28 [43]. An example item is “Been satisfied with the way you’ve carried out your task?”. Items were scored on a 4-point Likert scale and higher scores identified higher levels of social functioning. For the current sample, the seven items were grouped into a factor whose Cronbach’ alpha was 0.78.

#### 2.2.2. Independent Variables: Stress, Coping Styles, Self-Esteem, Social Support, Age, Educational Level, Number of Children and Marital Status

Stress was assessed by having recourse to two questionnaires: Life Events Questionnaire [46] and Chronic Stress Questionnaire [47]. The version for the elderly of the Life Events Questionnaire [46] consists of 19 items indicating the presence, during the previous 12 months, of life events and changes in the family, couple, economic, friends and health areas (i.e., “Death of a relative”). The chronic stress questionnaire is an open-response instrument in which participants provide information about the problems, conflicts and threats they currently face in their lives. Each answer is evaluated according to its severity, from 1 (little importance) to 3 (very important). The total score is obtained by adding the responses of the severity indicated in each problem and threats mentioned.

Coping styles were assessed by using the Spanish version of the Coping Styles Questionnaire [48]. It consists of 44 items rated on a 4-point Likert-type scale ranging from 0 (never) to 3 (always). An example item is “Try to find out more information to help make a decision about things”. In the factorization of old age individuals, it tests 3 factors: rational coping style, which comprises 15 items; emotional coping style, formed by 15 items; and detachment/avoidance coping style, consisting of 14 items. For the current sample, the Cronbach’s alpha was 0.87 for rational, 0.82 for emotional, and 0.73 for detachment/avoidance coping style.

The Spanish version of the York Self-Esteem Inventory [49] was used to evaluate self-esteem. The inventory consists of 58 items, rated on a 4-point Likert scale ranging from 0 (never) to 3 (always), which reflect various evaluative self-domains, including personal, interpersonal, familial, achievement, physical attractiveness, and the degree of uncertainty across the domains. An example item is “I think of myself in a positive way”. Items are grouped into a second-order factor that measures global self-esteem [50]. For the current sample, the Cronbach’s alpha coefficient for the self-esteem factor was 0.95.

Social support was analyzed by drawing on the Social Support Scale [51]. This scale consists of 12 items, rated on a 4-point Likert scale ranging from 0 (never) to 3 (always), which gather information on the availability of people who can help in the emotional, economic, work, familiar and advice/guidance needs. An example item is “Someone who listens when you need to talk about your feelings”. Items are structured in two factors: Emotional social support, consisting of 7 items, and instrumental social support, formed by 5 items. In the present sample, the Cronbach alpha for the emotional social support factor was 0.88 and for the instrumental social support was 0.81. The sociodemographic factors were gathered through a sociodemographic information collection sheet, whose main results are shown in Table 1.

### 2.3. Procedure

The participants in the study were volunteers, and were not remunerated for their participation. Tests were individually completed after reported consent was obtained; no names or any other data identifying the participant were used in the tests. We have complied with American Psychological Association ethical standards in the treatment of the sample. Access to the participants was through retirees’ association centers, as well as resorting to the social net of psychology and sociology university students trained in administering those tests, who received course credits for that task. This study forms part of an extensive research on gender and health and was positively evaluated by the Ethics Committee on Animal Research and Well-Being of the University of La Laguna (study approval number 2015-0170).

### 2.4. Statistical Analysis

Descriptive statistics were conducted to examine women’s and men’s sociodemographic characteristics. Comparisons between women and men were computed drawing on Pearson’s chi square test in case of categorical variables and by using *t*-test when they were continuous. The effect size of the mean differences was computed by using the Cohen’s *d*. The internal consistency reliability for the psychological distress, social functioning, coping styles, self-esteem and social support factors was calculated using the Cronbach’s alpha coefficient.

Hierarchical multiple regression analyses were performed to determine the relevance of the sociodemographic variables, stress, coping styles, self-esteem and social support in the psychological distress and in the social functioning of women and men. Logarithmic transformations were used on psychological distress to reduce skewness. In each regression analysis, sociodemographic variables were included in step 1. The age and number of children were considered continuous variables, and the level of studies an ordinal variable with 7 levels, as shown in Table 1. Scores were assigned from 0 (for without studies) to 6 (for 5-year university degree), so high scores indicate a higher educational level. Marital status was included as a dummy variable with two levels: one included married or living with a partner (reference category, which was coded with 0) and in the other the never married, separated, divorced, and widows, which was coded with 1. At step 2, the scores for the number of life events and chronic stress were incorporated. At step 3, coping styles were added to. And self-esteem and emotional and instrumental social support were included at step 4. The criterion considered was the score in psychological distress in the first regression analysis and the score in social functioning in the second. In this regression analyses the psychological distress score was also included at step 2, after the scores for the number of life events and chronic stress. Statistical analyses were conducted using the software IBM SPSS Statistics for Windows, version 21.0 (IBM Corp., Armonk, NY, USA).

## 3. Results 

### 3.1. Gender Differences in Psychological Distress, Social Functioning, Stress, Coping Styles, Self-Esteem and Social Support

Table 2 displays the means, standard deviations, and comparison between men and women in psychological distress, social functioning, stress, coping styles, self-esteem and social support. Statistically significant differences were found in six of the 10 variables, although the effect size of most of the differences was small. Women scored higher than men in psychological distress, chronic stress, emotional coping style, and instrumental social support. Men scored higher in self-esteem and rational coping style. No statistically significant differences were found between women and men in social functioning, the number of life events experienced during the previous year, detachment/avoidance coping style, and emotional social support.

Although there were no statistically significant differences between women and men in the number of life events experienced during the previous 12 months, there were differences in the frequency with which they cited four of the 19 events included in the questionnaire: serious illness of a family member, which was cited by 27.5% of men and 35.5% of women, χ^2^ (1, *N* = 1273) = 9.39, *p* = 0.002; death of a friend or intimate relationship, cited by 32.9% of men and 27% of women, χ^2^ (1, *N* = 1273) = 5.25, *p* = 0.02; change in working conditions, cited by 6.6% of men and 3.5% of women, χ^2^ (1, *N* = 1273) = 6.50, *p* = 0.01; and separation by geographical circumstances, cited by 2% of men and 4.5% of women, χ^2^ (1, *N* = 1273) = 5.25, *p* = 0.02. The most frequently cited events were: death of a relative, experienced by 35% of the sample, serious illness of a family member (31.8%), death of a friend or intimate relationship (29.8%), serious illness of a friend (23.1%), family discussions (21.1%), and debts (17.7%).

### 3.2. Predictors of Women’s and Men’s Psychological Distress 

Table 3 shows the main results of the hierarchical regression with the logarithm of psychological distress as the dependent variable for the men group, and Table 4 for the women group. *R*s were in both groups significantly different from zero at the end of each step. The sociodemographic variables entered into step 1 explained 1% of the variance in psychological distress in the men group and 5% in the women group. The change in *R*^2^ from model 1 to model 2 made clear that stress plays a significant role in men’s and women’s psychological distress. The addition of coping styles in model 3 resulted in an important increment in *R*^2^. The introduction of self-esteem and social support (model 4) also yielded a statistically significant increment in *R*^2^. Model 4, with all independent variables (IVs) in the equation, accounted for a total of 34% of the variance in psychological distress in both men and women. 

**Figure 1:**
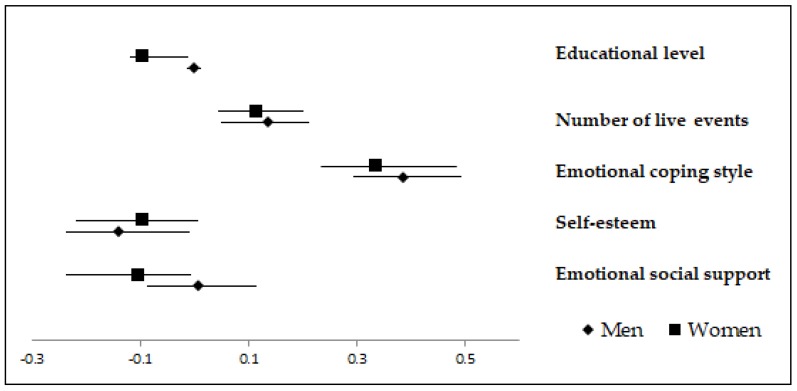
Beta values (95% CI) in the final model for men and women in the regression predicting psychological distress.

Beta values in model 4 showed that emotional coping style was the variable most associated with psychological distress for both genders, as men and women with greater emotional coping style reported greater psychological distress. In the men group self-esteem and the number of life events experienced during the last year were also significant predictors of psychological distress, so men with lower self-esteem and those who had gone through a larger number of life events during the last year reported greater distress. In the women group other significant predictors of psychological distress were the number of life events experienced during the last year, emotional social support, and educational level; thus, women who presented greater distress had experienced a higher number of events, counted on less emotional social support, and had a lower educational level. Figure 1 shows the Beta values for men and women obtained in the final regression model, which includes all IVs in the equation predicting the logarithm of psychological distress. This figure only includes the variables whose Beta values were statistically significant in women and/or men. 

### 3.3. Predictors of Women’s and Men’s Social Functionig 

Table 5 provides the main results of the hierarchical regression with social functioning as the dependent variable for the men group, whereas results from Table 6 correspond to the women group. *R*s were in both groups significantly different from zero at the end of each step. The sociodemographic variables entered into step 1 explained 3% of the variance in social functioning scores in the men group and 4% in the women group. The change in *R*^2^ from model 1 to model 2 made clear that stress and psychological distress play an important role in men’s and women’s social functioning (*R*^2^ change = 0.30 for the men group and *R*^2^ change = 0.29 for the women group). In the men group, the addition of coping styles (model 3) and self-esteem and social support (model 4) to the equation did not reliably improved *R*^2^. The final model (model 4) accounted for a total of 33% of the variance in social functioning in the men group and 35% in the women group. Beta values in model 4 identified that psychological distress was the variable most associated with social functioning for both genders, with better social functioning in men and women suffering from less psychological distress. Another significant predictor for both genders was age, with better social functioning in younger women and men. In addition, in the case of women self-esteem was also a significant predictor of social functioning, which was better in women with higher self-esteem. 

**Figure 2:**
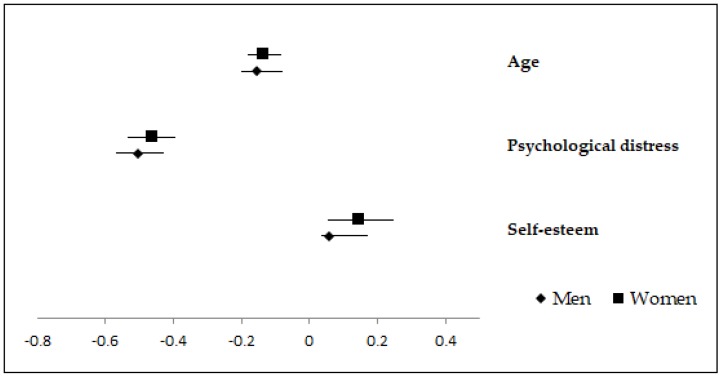
Beta values (95% CI) in the final model for men and women in the regression predicting social functioning.

Figure 2 displays the Beta values for women and men obtained in the final regression model, which includes all IVs in the equation predicting social functioning. This figure only records the variables whose Beta values were statistically significant in the case of women and/or men. 

## 4. Discussion

The present study has identified the relevance of psychological distress in the social functioning of the elderly, which improved significantly when people reported less psychological distress. In addition, social functioning was associated in older people with younger age. Although it had been hypothesized that greater stress, greater psychological distress and greater age would be related to worse social functioning, the results have identified that stress did not have a direct significant role in the social functioning of older men and women; nor did emotion-focused coping styles, social support and the number of children. Seemingly, educational level, marital status, self-esteem and rational coping style would neither play any important role in the case of men. In the women group, high self-esteem entailed better social functioning. In addition, a higher educational level and being married or living as a partner were associated with greater social functioning just in case the sociodemographic variables were included in the regression; yet this effect disappeared when stress and psychological distress were entered as predictors. 

The results of this work converge and extend the existing literature regarding the risk factor that psychological distress entails for the quality of life of the elderly [23,24,25,26]; moreover, the results of this work extend the knowledge and document that psychological distress also involves an important threat to the social functioning of old people.

The second hypothesis predicted that greater stress would associate with greater psychological distress; the results found in this study support this hypothesis, although regression analysis identified that chronic stress was no longer statistically associated to psychological distress when stress coping styles were included in the regression analysis. Other studies had revealed associations between stress and psychological distress [5,6,33,37,52,53], but although some researchers had considered chronic stress as a major threat to the health of the elderly [8], in the present study the number of life events experienced during the previous 12 months was a more important predictor of psychological distress than chronic stress. It may be owing to the fact that the most frequent life events cited by people in the present study sample were the illness and death of family members and loved ones, those events being initially less susceptible to coping by problem-focused coping styles than chronic stress.

In both genders the most noteworthy variable in psychological distress was the emotional coping style; these results are consistent with those yielded by another study conducted in Spain with people aged between 18–65 years old [36]. Less problem-focused coping was also related in both genders to greater psychological distress, although this association was no longer statistically significant when self-esteem and social support were included in the regression equation. The resulting findings are therefore consistent with those provided by other studies reporting that problem-focused coping was associated to less distress whereas emotion-focused coping was associated with greater distress [36,39,40,41]. In addition, lower self-esteem was a significant variable in men’s psychological distress, whereas, for women, it had to do with less emotional support and a lower educational level. These results cohere with those identified by other studies [18,27,28,33,34] that reveal that a high social support, self-esteem and educational level played a protective role against psychological distress, although this work has also made evident that the relevance of such factors differs in women and men. Such differences are congruent with gender stereotypes and traditional socialization patterns. In this sense, agency is central to men, who are characterized by focusing on the self and orient towards independence and the achievement of personal goals; communion is central to women, more oriented to other people and toward forming connections [54].

The first hypothesis, which predicted that women would have greater psychological distress than men, has been supported. These findings support those previously found in another research [28,32,34,36,39]. In addition, and in line with other studies conducted with younger people, it has been found that women display greater chronic stress than men, that their coping style is more emotional and less rational [36,39], and that their self-esteem scores lower [55]. However, although women’s psychological distress was rated higher than in the case of men, their stress coping style being less healthy and their self-esteem lower, the present study has revealed no differences between women and men in terms of social functioning. This may indicate the presence of protective factors in women which have not yet been identified, a task that should be addressed in future work.

Although the results of the present work allow to advance in the knowledge of the factors that are relevant in the active ageing of women and men, as well as in the risk and protective factors of such ageing, it presents some limitations. First, this is a cross-sectional study, therefore, it can only test the association between variables but not cause-effect relationships. Second, the sample, although large, has not been obtained through random sampling. The fact that people participated voluntarily in the study may imply some kind of bias since participants were older people who might have presented greater health and/or social functioning. Third, all the measures were self-report, so they may be influenced by social desirability. Fourth, all the participants lived in Spain, which may limit the generalization of the results with respect to other countries. Fifth, only a little more than a third of the variance in psychological distress and social functioning has been explained. Future research is needed to investigate the causal link between the variables, so as to increase awareness of the relevance of gender in active aging, to confirm the generalizability of these results in other countries, as well as to expand knowledge of the variables that determine the social functioning and psychological distress of elderly women and men.

## 5. Conclusions

Psychological distress was associated with worse social functioning in the elderly, which turned out to be lower as people aged. Gender is an important factor in the successful aging of the population, so it should be taken into account both in research and in programs and strategies aimed at achieving active ageing for all people.

## Figures and Tables

**Table 1:** Demographic characteristics of the male and female groups.

Variables	Men (*n* = 589)		Women (*n* = 684)		*χ^2^*-Value
	*n*	%	*n*	%	
Education					
Without studies	96	16.3	120	17.5	38.86 *
Elementary studies	231	39.2	331	48.4	
First grade professional training	24	4.1	30	4.4	
High school degree	110	18.7	79	11.5	
Second grade professional training	15	2.5	14	2.0	
3-year university degree	47	8.0	78	11.4	
5-year university degree	66	11.2	32	4.7	
Marital status					
Never married	33	5.6	40	5.8	96.85 *
Married/cohabiting	464	78.8	378	55.3	
Separated/divorced	36	6.1	55	8.0	
Widowed	56	9.5	211	30.8	
	**M**	**SD**	**M**	**SD**	***t*-Value**
Age	71.27	5.72	71.57	5.89	−0.93
Number of children	2.47	1.51	2.62	1.54	−1.71

* *p* < 0.001.

**Table 2:** Means (M), standard deviations (SD) and comparisons for males and females for the study variables.

Study Variables	Men (*n* = 589)		Women (*n* = 684)		*t*_(1271)_	*d*-Value
	M	SD	M	SD		
Psychological distress	11.56	8.05	13.87	8.73	−4.92 *	−0.27
Social functioning	13.42	2.20	13.23	2.57	1.45	0.08
Number of life events	1.94	1.50	1.97	1.58	−0.39	−0.01
Chronic stress	3.66	2.99	4.26	3.17	−3.42 **	−0.19
Rational coping style	26.36	7.70	24.91	7.74	3.33 **	0.19
Emotional coping style	12.98	6.30	15.02	6.78	−5.53 *	−0.31
Detachment/avoidance coping style	16.69	5.37	17.20	5.90	−1.60	−0.09
Self-esteem	113.96	20.86	109.00	22.61	4.07 *	0.23
Emotional social support	16.59	4.34	16.46	4.46	0.56	0.03
Instrumental social support	9.42	3.86	9.92	3.82	−2.29 ***	−0.13

* *p* < 0.001; ** *p* < 0.01; *** *p* < 0.05.

**Table 3:** Summary of the hierarchical regression with psychological distress as the dependent variable for the men group.

Variables	Model 1		Model 2		Model 3		Model 4	
	*β*	*t*-Value	*β*	*t*-Value	*β*	*t*-Value	*β*	*t*-Value
Age	−0.03	−0.55	−0.00	−0.09	−0.05	−1.26	−0.04	−1.02
Educational level	−0.13	−3.07 **	−0.10	−2.39 ***	0.01	0.20	−0.00	−0.00
Number of children	−0.02	−0.46	−0.04	0.86	−0.02	−0.49	−0.01	−0.31
Never married/separated divorced/widowed	0.01	0.19	−0.03	−0.61	−0.03	−0.93	−0.04	−1.11
Number of life events			0.26	6.29 *	0.14	3.89 *	0.14	3.84 *
Chronic stress			0.08	2.01 **	0.05	1.33	0.04	1.06
Rational coping style					−0.13	−3.18 **	−0.06	−1.41
Emotional coping style					0.48	12.42 *	0.39	7.66 *
Detachment/avoidance coping style					−0.06	−1.50	−0.06	−1.50
Self-esteem							−0.14	−2.50 ***
Emotional social support							0.01	−0.14
Instrumental social support							−0.07	−1.52
*R* ^2^	0.02		0.09		0.34		0.36	
Adjusted *R*^2^	0.01		0.08		0.33		0.34	
*R*^2^ Change	0.02		0.08		0.25		0.01	
ANOVA (*F*-value, df)	2.40(4) ***		10.01(6) *		33.54(9) *		26.37(12) *	

*β* = Standardized regression coefficient. *R*^2^ = explained variance. * *p* < 0.001; ** *p* < 0.01; *** *p* < 0.05.

**Table 4:** Summary of the hierarchical regression with psychological distress as the dependent variable for the women group.

Variables	Model 1		Model 2		Model 3		Model 4	
	*β*	*t*-Value	*β*	*t*-Value	*β*	*t*-Value	*β*	*t*-Value
Age	−0.08	−1.89	−0.05	−1.18	−0.06	−1.79	−0.04	−1.16
Educational level	−0.21	−5.25 *	−0.20	−5.24 *	−0.08	2.14 ***	−0.10	−2.74 **
Number of children	0.01	−0.32	−0.01	0.33	−0.01	−0.16	0.01	0.31
Never married/separated divorced/widowed	0.10	2.63 **	0.08	2.23 ***	0.04	1.27	0.03	1.02
Number of life events			0.19	5.23 *	0.12	3.65 *	0.11	3.47 **
Chronic stress			0.16	4.19 *	0.05	1.51	0.05	1.37
Rational coping style					−0.11	−2.76 **	−0.04	−0.80
Emotional coping style					0.43	11.54 *	0.34	7.21 *
Detachment/avoidance coping style					−0.06	−1.70	−0.06	−1.66
Self-esteem							−0.09	−1.82
Emotional social support							−0.10	−2.10 ***
Instrumental social support							−0.06	−1.17
*R* ^2^	0.05		0.12		0.33		0.35	
Adjusted *R*^2^	0.05		0.12		0.32		0.34	
*R*^2^ Change	0.05		0.07		0.20		0.02	
ANOVA (*F*-value, df)	8.99(4) ***		15.91(6) *		36.41(9) *		30.22(12) *	

*β* = Standardized regression coefficient. *R*^2^ = explained variance. * *p* < 0.001; ** *p* < 0.01; *** *p* < 0.05.

**Table 5:** Summary of the hierarchical regression with social functioning as the dependent variable for the men group.

Variables	Model 1		Model 2		Model 3		Model 4	
	*β*	*t*-Value	*β*	*t*-Value	*β*	*t*-Value	*β*	*t*-Value
Age	−0.16	−3.50 **	−0.15	−4.09 *	−0.15	−3.90 *	−0.15	−4.05 *
Educational level	0.06	1.44	−0.01	−0.19	−0.02	−0.53	−0.02	−0.41
Number of children	0.08	1.66	0.06	1.52	0.06	1.41	0.05	1.31
Never married/separated divorced/widowed	−0.07	−1.60	−0.04	−1.23	−0.04	−1.15	−0.03	−0.83
Number of life events			−0.01	−0.36	−0.01	−0.35	−0.01	−0.29
Chronic stress			−0.03	−0.85	−0.03	−0.76	−0.02	−0.65
Psychological distress			−0.54	−15.27 *	−0.51	−12.08 *	−0.50	−11.83 *
Rational coping style					0.05	1.19	0.01	0.27
Emotional coping style					−0.03	−0.67	0.02	0.28
Detachment/avoidance coping style					0.05	1.35	0.05	1.31
Self-esteem							0.06	1.02
Emotional social support							0.06	1.01
Instrumental social support							0.01	0.20
*R* ^2^	0.03		0.33		0.34		0.34	
Adjusted *R*^2^	0.03		0.33		0.33		0.33	
*R*^2^ Change	0.03		0.30		0.01		0.01	
ANOVA (*F*-value, df)	5.19(4) *		41.50(7) *		29.71(10) *		23.22(13) *	

*β* = Standardized regression coefficient. *R*^2^ = explained variance. * *p* < 0.001; ** *p* < 0.01.

**Table 6:** Summary of the hierarchical regression with social functioning as the dependent variable for the women group.

Variables	Model 1		Model 2		Model 3		Model 4	
	*β*	*t*-Value	*β*	*t*-Value	*β*	*t*-Value	*β*	*t*-Value
Age	−0.09	−2.32 ***	−0.14	−4.12 *	−0.13	−3.78 *	−0.14	−4.04 *
Educational level	0.11	2.85 **	0.02	0.47	0.01	−0.34	−0.01	−0.13
Number of children	−0.06	1.43	−0.03	−1.02	−0.04	−1.06	−0.04	1.16
Never married/separated divorced/widowed	−0.08	−2.09 ***	−0.02	−0.66	−0.02	−0.57	−0.01	−0.44
Number of life events			0.03	0.96	0.04	1.11	0.04	1.09
Chronic stress			−0.06	−1.81	−0.04	−1.32	−0.04	−1.19
Psychological distress			−0.54	−15.92 *	−0.47	−12.38 *	−0.46	−12.01 *
Rational coping style					0.11	2.80 **	0.05	1.03
Emotional coping style					−0.07	−1.65	0.01	0.22
Detachment/avoidance coping style					0.01	0.34	0.01	0.37
Self-esteem							0.14	2.74 **
Emotional social support							−0.07	−1.35
Instrumental social support							0.09	1.95
*R* ^2^	0.05		0.33		0.35		0.36	
Adjusted *R*^2^	0.04		0.32		0.34		0.35	
*R*^2^ Change	0.05		0.29		0.02		0.01	
ANOVA (*F*-value, df)	8.08(4) *		47.70(7) *		35.97(10) *		28.85(13) *	

*β* = Standardized regression coefficient. *R*^2^ = explained variance. * *p* < 0.001; ** *p* < 0.01; *** *p* < 0.05.
